# Alcohol marketing on YouTube: exploratory analysis of content adaptation to enhance user engagement in different national contexts

**DOI:** 10.1186/s12889-018-5035-3

**Published:** 2018-01-16

**Authors:** Himanshu Gupta, Tina Lam, Simone Pettigrew, Robert J. Tait

**Affiliations:** 1 National Drug Research Institute, Faculty of Health Sciences, Curtin University, Perth, WA 6008 Australia; 2 School of Psychology and Speech Pathology, Faculty of Health Sciences, Curtin University, Perth, WA 6008 Australia

**Keywords:** Alcohol, Marketing, Internet, Social media, YouTube, India, Australia

## Abstract

**Background:**

We know little about how social media alcohol marketing is utilized for alcohol promotion in different national contexts. There does not appear to be any academic work on online exposure to alcohol marketing via social media in India, and most of the limited research in Australia has focused on Facebook. Hence, the present study extends previous research by investigating alcohol promotion conducted on an under-researched form of social media (YouTube) in two contrasting geographic contexts. This study examines and compares the types of strategies used by marketers on Indian and Australian alcohol brands with the greatest YouTube presence, and the extent to which users engage with these strategies.

**Methods:**

The 10 alcohol brands per country with the greatest YouTube presence were identified based on the number of ‘subscriptions’. The number of videos, views per video, and the type of content within the videos were collected for each brand. The data were analyzed using an inductive coding approach, using NVivo 10.

**Results:**

The targeted brands had gathered 98,881 subscriptions (Indian brands: *n* = 13,868; Australian brands: *n* = 85,013). The type of marketing strategies utilized by brands were a mix of those that differed by country (e.g. sexually suggestive content in India and posts related to the brand’s tradition or heritage in Australia) and generic approaches (e.g. encouraging time- and event-specific drinking; demonstrations of food/cocktail recipes; camaraderie; competitions and prize draws; and brand sponsorship at music, sports, and fashion events).

**Conclusions:**

This cross-national comparison demonstrates that YouTube provides alcohol marketers with an advertising platform where they utilize tailored marketing approaches to cater to specific national contexts and develop content on the cultural meanings users invoke in their interactions with these strategies. Those exposed to alcohol marketing on YouTube are likely to include those under the legal drinking age.

**Electronic supplementary material:**

The online version of this article (10.1186/s12889-018-5035-3) contains supplementary material, which is available to authorized users.

## Background

Along with conventional marketing media such as television and print, alcohol marketers have adopted social media platforms as contemporary marketing tools. As well as influencing alcohol use [1, 2], online conversations about alcohol are postulated to become central to identity construction processes, development, and maintenance of relationships and lifestyles [3]. These intertwined processes of identity construction and influence make social media a powerful medium for alcohol marketers to create and effectuate strategies to target audiences, including young people [4–6].

Alcohol marketing on social networking sites (SNS) is different to traditional media in the way that brands encourage SNS users to co-produce content and leverage users’ individual identities and their social relationships as reflected in the content they post on their SNS profiles [7–9]. As per the algorithm predictions of SNS, alcohol marketing on SNS commences with brands instigating, interacting, and observing the communication processes between brands, users, and users’ online peer network [10]. This is followed by brands directing those conversations in real-time and embedding themselves within users’ lifestyles, identity-making processes, and cultural practices; and developing an active and continuous conduit facilitating the flow of apparently enjoyable peer-to-peer transmissions of marketers’ messages that are disseminated through users online networks [8, 11, 12]. Further, such online branded conversations could provide data on users and their online peer networks, which could facilitate future marketing to niche groups (e.g. young people). This new method of communicating with SNS users represents an enormous evolution from the use of traditional advertising media that can only communicate much more static representations of product meaning/symbolism.

It has been suggested that alcohol marketing on SNS facilitates identity construction among users [8]. Young people, in particular, interact with marketing content in a way that brands come to belong to their socio-cultural identities and lifestyles [13]. For example, interaction with brand sponsorship at cultural events and event-related marketing (e.g. competitions) on SNS are especially appealing to young people. This potentially makes them less critical of marketing techniques that attempt to integrate the brand content in their cultural spaces. Firstly, because they see such marketing as opportunities for ‘self-gain’ (e.g., win prizes, free entry to the events, and free alcohol), and secondly it provides them with an opportunity for self-representation and social acceptability via an event which has a pre-existing social currency (especially within their peer networks), both online and offline [8, 12].

These types of communication processes are also conducive to brands, for example, brands ask users to tag themselves in the brand content posted on SNS or check-in the event locations on their SNS profiles. This facilitates the flow of content into the users’ SNS peer networks, enhancing further creation of user-generated content [8]. Hence, embedding marketing content into users’ leisure, peer networks, and cultural spaces is one of the vital factors in influencing ‘brand-user interaction’ on SNS [14].

As occurs with other social media platforms [15], alcohol marketers are likely utilizing tailored marketing approaches on YouTube to engage users. This tailoring can occur at the cultural level, hence a cross-national comparison of YouTube alcohol brands’ sites has the potential to explore how alcohol marketers use social media platforms to adapt their online promotional activities to specific cultural contexts. A comparative approach was adopted for this study using India and Australia as contrasting cultural contexts, representing one of the first attempts to comprehensively analyze different approaches to alcohol marketing on YouTube across countries.

### Drinking prevalence and patterns

Historically and socio-culturally, India and Australia differ substantially and have contrasting drinking cultures. The annual per capita consumption of pure alcohol is estimated at 4.3 L in India [16] compared to 9.7 l in Australia [17]. Nevertheless, the active marketing of alcohol in India (and China) is thought to have driven the recent global increase in alcohol use [16], emphasizing the importance of exploring alcohol marketing strategies utilized by alcohol companies in this rapidly changing country. In both countries, men drink more, but there is a greater gender disparity in India. Indian women drink 6% (0.5 L) of their male counterparts’ alcohol volume (8 L), while Australian women consume 42% (7 L) of their male counterparts (17 L) [16]. Furthermore, in India, alcohol use is more prevalent in the lowest wealth quintile than in the highest wealth quintile and among those with lower levels of education [18]. In contrast, in Australia, those in the top wealth quintile and those with more education have a higher prevalence of drinking [19].

There are a multitude of factors that potentially contribute to the consumption pattern differences between India and Australia. For example, in India, there are socio-cultural norms that are less accepting of alcohol [20], proscriptions of commonly practiced religions (e.g. lower prevalence of drinking among the 14% of Indians who identify as Muslims) [20, 21], and with the legal drinking age in India ranging from 18 to 25 years, with complete prohibition in certain states [22]. Given the importance of market segmentation in marketing strategies [15], these factors are likely to result in different marketing strategies being produced by alcohol brands between the two countries to optimize sales.

### Regulatory environment

Different countries have different alcohol advertising regulations. In India, alcohol advertising regulation is subject to the Advertising Standard Council of India [23]. Although ASCI imposes a blanket ban on alcohol advertising in traditional media; alcohol marketers manage to promote their brands through surrogate advertising. Examples include using the same alcohol brand name on non-alcoholic products such as merchandise; brand sponsorship at sports, music, and fashion events; and celebrity endorsement. However, this Code does not prohibit digital alcohol advertising, so advertising via social media remains unfettered and is extensive in India [22].

In Australia, alcohol advertising is industry-regulated via the Alcohol Beverages Advertising Code (ABAC). This Code applies to all marketing communications (including digital media) in Australia. As per this Code, “a marketing communication must not show (visibly, audibly or by direct implication) the consumption or presence of an Alcohol Beverage as a cause of or contributing to the achievement of personal, business, social, sporting, sexual or other success” (ABAC, 2013, p.2) [24]. It further restricts the depiction of content that has strong or evident appeal to minors (e.g., imagery and cartoon characters), applying brand extensions (e.g., logos on merchandise) to non-alcohol beverage products, and the placement of content in digital media where there are no age-restrictions [24]. However, this Code primarily relates to content, although restrictions on placement apply to outdoor advertisements and commercials on free-to-air television. Hence, digital media platforms are conducive to alcohol promotion in Australia.

In July 2017, ABAC expanded their Code to include placement standards, for example, to only use media platforms with a 75% adult audience. It is of note that even the most youth friendly platforms would struggle to have >25% of their audience being within a 5 year age bracket (13–17 year olds), I.e. despite the new placement restriction including social media, the ‘75% adult’ criterion means it is in effect, the same as no restriction at all on SNS [25].

### Research significance

YouTube is used extensively, with an estimated 40 million YouTube users in India [26], with about 70% of them below 35 years of age [27]. In Australia, this is estimated at 14 million [28], with about 51% of Australian teenagers (aged 14–17 years) were reported using YouTube in 2013 [29]. While we identified only one academic study that had investigated social media alcohol marketing in the Indian context [15]; several such studies were identified for Australia. However, the limited Australian work had primarily examined alcohol brand content posted on Facebook [12–14, 30, 31]. Several international studies have reported the types of marketing strategies alcohol companies use to market their products on SNS. The strategies include the frequent use of cartoon characters; real-world tie-ins; interactive games; competitions; time-specific suggestions to drink; comedy; engagement with fashion, music, and sporting events; engagement with local venue and event marketing; references to brand heritage; memes; and sexual imagery [8, 9, 32]. Little is known about how marketers engage users with alcohol brand content on YouTube, with just two studies identified. Cranwell et al. (2017) found positive associations between viewing YouTube music videos and alcohol use among 11–18 years old British adolescents [4]. The content of these videos included sexualized imagery or lyrics, associating alcohol use with one’s image, lifestyle, and sociability, and content explicitly encouraging excessive drinking. Barry et al. (2015) found 67% of the YouTube alcohol brands’ channels were successfully viewed by underage American adolescents, circumventing age-restriction measures [33].

There is evidence that exposure to online alcohol marketing increases the likelihood of alcohol use [1, 30] and alcohol-related problems and increased risk of developing alcohol use disorders [34], especially among youth [30]. It has also been demonstrated that alcohol marketers use tailored marketing strategies to cater to specific national contexts [15]. The present study extends previous research by investigating alcohol promotion conducted on an under-researched form of social media (YouTube) in new geographic contexts (India and Australia). We aimed to provide insight into the marketing strategies used by alcohol companies to promote their products in culturally diverse locations and the extent of user engagement on official YouTube channels. This information is important to guide both national and international efforts to minimize harmful alcohol consumption resulting from exposure to online alcohol marketing, especially among young people.

## Methods

### Sampling and data collection

The search strategy was informed by two recent studies. One investigated both brand- and user-generated alcohol-related content posted on Indian and Australian alcohol brands’ Facebook pages [15] and the other examined content posted by alcohol companies and users’ responses to that content posted on Australian alcohol brands’ Facebook pages [14]. Initially, we compiled a comprehensive list of alcohol brands distributed in India [22, 35] and Australia [36, 37]. These comprised 256 Indian and 287 Australian brands. We searched for each brand’s YouTube page.

YouTube is a global social media channel. However, YouTube pages may be country-specific, but accessible from around the world. To confirm if the brand had a relevant Indian/Australian YouTube page, we either sought a statement such as ‘this is the official country page’ on the brand’s YouTube page or accessed the brand’s national official website and searched for a hyperlink to their national YouTube page. The absence of a national official YouTube page excluded the brand from the analysis. We found 12 Indian and 42 Australian eligible YouTube pages (Table 1).

**Table 1:** Search strategy to select the top 20 alcohol brands with the greatest YouTube presence, 10 each for India and Australia

	India	Australia
Alcohol reports used to identify alcohol brands active in each country	• Report on alcohol marketing and regulatory policy environment in India (2013) • Liquor Brands in India (2013)	• MCAAY (2014) • IBIS World Industry Report Beer Manufacturing in Australia (2015) • IBIS World Industry Report Spirit Manufacturing in Australia (2015)
No. of alcohol brands distributed in the country	256	287
Brands with an unofficial YouTube presence	12	42
Brands with dedicated official YouTube, pages	11	33

Finally, the 10 brands in each country with the highest number of subscriptions were selected for analysis (notably, this may not reflect the brands’ market share). There was no overlap between the top ten brands identified for the two countries but Pernod Ricard and Bacardi own multiple Indian and Australian brands included in the study (Additional file 1).

For each brand we extracted year the YouTube page was started, numbers of subscriptions, videos, and views per video. In this study, we defined engagement to include users either viewing or commenting on material posted on brands’ pages. Links to the brands’ official website, Facebook, Google+, information on messages relating to responsible drinking and legal drinking age were also collected. These publically available data were retrieved for a period of 2 months: February 1 to March 31, 2016. Approval to access these data was obtained from the University’s Human Research Ethics Committee, with assurances that personal identifying information would not be revealed.

### Coding process and analysis

As YouTube offers video-based content, videos available on a brand’s YouTube page were watched and examined and the content was analyzed using NVivo10. The objective was to categorize themes shown in the videos. An overview of the coding process has been presented in Table 2. Due to the use of an emergent coding process, a single coder analyzed the data to develop new codes to reflect the content of the data. Although the coding process started with the development of a list of a priori codes (e.g. competitions; time-and-event specific suggestions to drink; comedy; and fashion, music, and sporting events) identified from the relevant alcohol literature [6, 8, 9, 14, 32], new patterns and themes emerging from the raw data were identified (e.g. celebrity endorsements, gender-specific posts, and inspirational talks). As occurs with an inductive (thematic) coding process, we intended to create as few categories (themes) as possible [38–40]. This process facilitated capturing key themes in the raw data and combining the smaller categories into more encompassing categories in consultation with the research objectives [41]. The resulting NVivo nodes were investigated to generate themes, which were subsequently discussed among research team members to refine the final themes. This method has previously been used to analyze themes from alcohol companies’ Facebook sites [15].

**Table 2:**
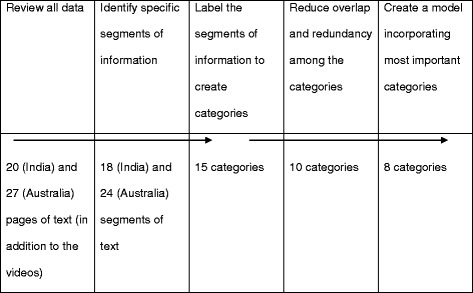
The coding process in inductive analysis (adapted from Creswell, 2002, Fig. 9.4, p266) [56]

## Results

### User engagement

Additional file 1 shows the 10 leading brands for India and Australia with their descriptive data. The majority of the Indian brands established YouTube pages between 2010 and 2015, while the start dates for Australian brands ranged from 2005 to 2010. Of the Indian brands there were: five whiskies (McDowell’s No. 1, Blenders Pride, Haywards 5000, Officer’s Choice, and Ricard), two beers (Kingfisher and Foster’s), one vodka (White Mischief), one rum (Bacardi), and one ready-to-drink (Breezer; rum-based) beverage. In contrast, the Australian brands comprised four beers (XXXX, Corona Extra, Coopers Ale, and Carlton Draught), two whiskies (Jameson Irish Whiskey and Jack Daniel’s), and one each for wine (Jacob’s Creek), gin (Bombay Sapphire), rum (Bundaberg Rum), and a vodka (Absolut) product. The brands with the highest number of subscriptions were whisky brands in both countries (India: McDowell’s No. 1; Australia: Jameson Irish Whiskey).

The brands had a total of 98,881 subscriptions and 100,718,549 views. Australian brands accrued greater user engagement than their Indian counterparts. For example, Jameson Irish Whiskey generated 31,927,010 views, compared with 34,712,801 for the 10 Indian brands. The number of views and videos was directly proportional to the number of subscriptions for the majority of the Indian brands. In comparison, Australian brands did not demonstrate any such trend with marked differences in the rank ordering by subscriptions or views. The number of subscriptions gives the best available indication of the actual reach of YouTube among users [9, 32]; however this is not a measure of the number of people who may have actively viewed each video. The numbers reported in Additional file 1 reflect the cumulative numbers in each category ever posted on a brand’s YouTube page by users or brands. Further, the majority of the Indian and Australian brands’ pages had links to Facebook and Google+, increasing the reach of these brands to other popular social media channels. Although considerable variance in user engagement and brand popularity is identified, alcohol companies appear to utilize similar marketing strategies and themes, across SNS, with within and between country differences [15].

### Strategies utilized for alcohol marketing

Brands posted a variety of content on their YouTube pages to engage users (Additional file 2). The strategies employed were a mix of those differing by country (e.g. India: sexually suggestive content vs Australia: posts related to the brand’s tradition or heritage) and generic approaches (e.g. encouraging time- and event-specific drinking (TESD); demonstrations of food/cocktail recipes; camaraderie; competitions and prize draws; and brand sponsorship at music, sports, and fashion events). Additional file 3 shows the frequency of references to the content for each category.

A. Common strategies

#### TESD

Fourteen brands included TESD references which encouraged drinking at a particular time or event. Common examples included after work, on weekends and public holidays, on special occasions (e.g. St. Patrick’s Day), and when traveling:

This summer, Foster's will make sure, no matter where you go, it'll always be DAAAAAMN COLD! Watch our latest TVC and get a taste of DAAAAAMN COLD REFRESHMENT (Foster’s, India).

My son, Bryan, and I don't wait for St. Patty's we take a nip every now and then, we have our own Jameson Glasses, which we prefer to the Chivas glasses, happy drinking to you (Jameson Irish Whiskey user comment, Australia).

Carlton Draught’s “Aussie pub tour videos” and brand-i Blue Mile Travelogues videos (Kingfisher, India) are among other TESD examples.

#### Consumption suggestions

Five brands (both Indian and Australian) posted videos demonstrating cocktail and/or food recipes. Some of the Indian brands (e.g. Breezer) linked their drinks to popular Indian flavors such as Aam Panna (mango drink) and Nimbu Pani (lemon drink). Users readily engaged with such posts by suggesting recipes and asking for more information:

Bombay, 7up, two lime wedges; pour, squeeze, stir, enjoy (Bombay Sapphire user comment, Australia).

Where is the recipe for the Sapphire Imperial Sun, winner of last year's contest? (Bombay Sapphire user comment, Australia).

References to camaraderie and togetherness were seen on the YouTube accounts of both Indian and Australian brands (four brands). While Jacob’s Creek (Australia) posted a friendship stories series titled “Sparkling around the world reunion” [with friends], Jameson Irish Whiskey (Australia) uploaded videos titled “Long live the neighbors” that featured stories about drinking buddies. Similarly, McDowell’s No.1 (India) uploaded videos titled “No1Yaari, an ode to YAARI [friendship]” in several Indian languages.

#### Brand sponsorship at music, fashion, and sporting events

Another common theme was the alcohol sponsorship of music, fashion, and sporting events (11 brands). Kingfisher’s (India) “Strong backstage music videos” featuring famous Indian singers performing in different Indian languages, Bacardi (India) sponsored music events such as “MH-7 weekender”, and Jack Daniel’s and Corona Extra’s sponsored music concerts are some of the examples. There were references to brand sponsorship of sports events, such as White Mischief (India) uploading videos featuring Indian and International cricket players celebrating success and Cooper’s Ale (Australia) promoting its sponsorship of surfing events. Brand sponsorship at fashion events was associated with Blender’s Pride (India), Officer’s Choice (India), and Carlton Draught (Australia).

#### Competitions and giveaways

Event-related competitions and prize draws were published by both Indian and Australian brands’ (six brands). McDowell’s No.1 (India) promoted multiple contests, such as the “Share a hug contest” that provided users with the opportunity to win a chance to meet their favorite cricket players. Other contests included “Karaoke world championship” and “Celebrate your yaari [friendship]” contest (McDowell’s No.1, India). Bacardi (India) organized a “Bacardi Music CDs legacy competition” that involved users posting their bartending videos for the chance to win up to US$1600. These kinds of posts received positive responses from users: “I would like to win please & I would [like] to attend mamma awards” (Absolut user comment, India).

References to inspirational talks were found for both Indian and Australian brands (three brands). Officer’s Choice Blue (India) used its “Raise your voice” campaign to promote equality across the strata of society. Jacob’s Creek (Australia) posted its “Made by Moment” film series, featuring a leading male tennis player, linking dreams, inspirations, and determination to success. Haywards 5000 (India), under its establishment, “Hausla Buland Academy”, offered “Hauslay ki Udaan”, a unique entrepreneurship program that focuses on inspiring, supporting, and mentoring young entrepreneurs to help bring their ideas to life. Users were provided with business ideas in different Indian languages via testimonials from previous beneficiaries of such programs and celebrities.

#### References to responsible drinking and legal drinking age

Messages relating to responsible drinking were found on five Australian and four Indian brands’ pages. Examples of such messages included “live freely, drink responsibly” (Jack Daniel’s, Australia), “Enjoy Jacob’s Creek responsibly” (Jacob’s Creek), and “We encourage drinking responsibly” (White Mischief, India). Pernord Ricard (Australia) also posted videos associated with responsible drinking and drinking and driving. However, only Bundaberg (Australia) had links to relevant health, government or to industry-supported sites (e.g. “DrinkWise”). References to the legal drinking age were even fewer. Three Indian and two Australian brands provided users with information such as “This page is for all Carlton Draught (Australia) fans over the age of 18 to enjoy responsibly” and “Please view videos and/or subscribe to the channel only if you're above legal drinking age in your region” (Kingfisher, India).

B. Strategies that differed by country

#### Brand tradition and heritage

Four Australian brands also posted videos portraying brands’ traditions and cultural heritage. For example, Jameson Irish Whiskey uploaded videos relating to whisky production. Bundaberg appeared to be attempting to align its content with the everyday conversations of working class males: “Men like us like Bundaberg rum”, “Men like us like craftsmanship”, “Men like us like rummanship”, and “Men like us like witmanship”. These posts can be interpreted as an attempt to enhance the brand’s authenticity and credibility by appealing to men by providing them with content related to Australian masculine identities. Such strategies were mainly associated with Australian brands rather than their Indian counterparts.

#### Sexually suggestive content (gender targeting)

Four Indian brands published posts containing sexually suggestive content. Kingfisher uploaded videos on the making of “KF swimsuit special calendars”, featuring attractive female models in swimming costumes, and Bacardi posted “Bacardi beach” videos with a similar theme. White Mischief posted images and videos of attractive women in revealing clothing lying by a pool and drinking vodka. The images included the tagline “We're naughty, we're fun and we're here to put you in the mood for mischief [wink emoticon]” and “Flirt with the Mischief Gals”. Other videos showed the brand’s female promotional staff asking questions to users and prompting them to ask questions in return. For example:

What do you think makes a good flirt? (Question from brand model).

Love this channel, you girls are so sexy. Can we have more videos of you performing please? (User post).

In contrast, suggestive content of this nature was not evident on the Australian brands’ YouTube pages. However, the above mentioned Bundaberg example shows that there is still some level of gender targeting by Australian brands.

Overall, brand activities on YouTube were considerable in terms of the number of subscriptions and views accrued, the frequency of references to the themes identified, and the timing of content posted (e.g. references to TESD). Brands leveraged interaction with users by initiating and responding to routine conversations about everyday life and creating real-world activities on YouTube (e.g. references to consumption suggestions, competitions, and entrepreneurship). This process facilitated co-creation of content, with user contributions being a key part of brand content. The user-generated content thus created was utilized by brands to further create and tailor content within users’ individual (e.g. displaying selective alcohol-related material based on users’ preferences, gender targeting), social (e.g. references to camaraderie/togetherness), and cultural spaces (e.g. country-specific event-based brand promotion and consumption suggestions). These strategies also appeared to be attempts to target niche groups, especially young people (such as through references to music and fashion events, competitions, giveaways, and inspirational talks) [8, 9].

## Discussion

There is evidence that exposure to online alcohol marketing increases the likelihood of alcohol use and subsequent alcohol-related problems [1, 3]. Thus, understanding online alcohol marketing strategies is an important prerequisite for policy changes and other interventions.

There are an estimated 432 million Internet users in India (5% of the total population). Of these, 51% (60% male) of urban and 48% (75% male) of rural Internet users use the Internet daily. Further, about 65% of Indian Internet users are below 25 years of age [42]. In contrast, Australia has about 23 million Internet users (95% of the total population), of whom 87% are daily Internet users (88% metropolitan; 85% regional). Among daily users, about 69% use SNS, with similar rates of use among males and females. About 75% of SNS users belong to the 18–29 years age group [43]. These numbers reflect ‘digital divides’ within countries based on age (more SNS users aged below 30 years), gender (more male SNS users in India), and location (more SNS users in urban locations) [42, 43]. Such divides potentially influence SNS use and thus involvement with alcohol marketing across social media platforms.

We found that although India has more YouTube users than Australia (40 million vs 14 million) [26, 28], the Australian brands demonstrated greater user engagement than their Indian equivalents. Of the total subscriptions for the top 20 brands across the two countries, 85% were for Australian brands. Most of the Australian brands established a YouTube presence earlier than the Indian brands, potentially indicating the relative maturity of the two markets. More speculatively, these differences may further suggest that marketers are moving from countries where there are stronger alcohol advertising regulations (e.g. Australia) to less regulated markets with greater growth potential (e.g. India).

Although the reasons for the lower level of user engagement for the Indian brands are unclear, these are likely to be an interplay of socio-cultural norms that are less accepting of alcohol [44], religious practices [20], the legal alcohol purchasing age in India which ranges from 18 to 25 years [22], and significant gender differences in alcohol consumption [16]. There are fewer cultural inhibitions to prevent users from engaging with brands in the Australian context, which is likely to account for the greater user engagement observed for the Australian brand pages.

The preference for spirits among Indian alcohol users [16, 22] and the popularity of whisky brands on Indian YouTube pages suggests that the Indian brands with the most subscribers on YouTube reflect and may reinforce local alcohol preferences. Similarly, the popularity of beer among Australians [45] possibly explains the presence of four beer brands among the top 10 Australian YouTube pages.

References to brand sponsorship of music, sports, and fashion events were seen in both the Indian and Australian context. While the marketers did not directly endorse heavy drinking at those events, they provided users with event-linked branded content that could be used to prompt or support everyday conversations about the sponsored events and thus possibly influence users to consume their products. Companies can promote their products through sponsorship because this is not covered under the existing Indian and Australian online alcohol advertising policies [5, 22].

Despite commonalities, marketing strategies differed by country. Notably, many of the Indian brands used sexually suggestive content, often including attractive female models. A lack of references to sexually suggestive content on Australian YouTube pages is potentially due to ABAC’s responsible depiction of alcohol guidelines that discourage the portrayal of alcohol contributing to sexual success [24]. Furthermore, as the average level of alcohol consumption among men is far greater than that of women in India [16], it is likely that Indian alcohol brands consciously target men by using female models in their online communications. In a country where sex is a taboo subject [46], and for example, it is socially unacceptable for women to wear bikinis in public, even in public swimming pools; this could be an easy way to attract young men to their YouTube sites and brands, potentially increasing the sales of their products. Nevertheless, as shown by the Bundaberg example, there was still gender targeting in Australia, albeit to a lesser extent. The Bundaberg posts explicitly celebrated masculine identities as typically associated with working class men to reinforce positively the validity of their pre-existing identities. In contrast, although the Indian brand Officer’s Choice also appeared to appeal to working class men, the content was mainly calling for the elevation of their class. India’s middle class is currently in a rapid growth phase, whereas Australia’s middle class has been relatively static [47]. These Indian advertisements appeared to be attempts to use alcohol as an indicator of social equality and progression, especially for the disadvantaged strata of society.

Alcohol marketing via YouTube in India and Australia also appears to be based on the existing interests of its consumers. For example, references to brands’ traditions or heritage (in the Australian context) appeared to be attempts to create stories relating to users’ traditions, interests, and values, hence localizing the brands, making them part of real communities, regions, and livelihoods, and integrating the consumption of products within those interests.

Further, instances of inspirational talks, featuring famous celebrities narrating their stories and linking them to success, in the presence of an alcoholic beverage in the videos, were identified on alcohol brand YouTube channels, across countries. Adolescents often associate celebrities with alcohol, glamor, attractiveness, and sexuality [48]. This likely results in adolescents having positive attitudes towards alcohol because they trust celebrities and have a tendency to imitate them, a process referred to as ‘wishful identification’ [49]. Roy et al. (2017) demonstrated that about half of all respondents (school students) were willing to follow advice from their favorite celebrity. They also suggested that such endorsements increase the likeability and credibility of the brands among users and potentially increase brand sales [50]. This is likely the reason that references to celebrity endorsement were seen for a few brands in both countries. In the context of regulation, this is especially pertinent for Australia as despite the self-regulatory Alcohol Beverage Advertising Code [24] specification for the responsible depiction of the effects of alcohol that includes not showing or directly implying the consumption or presence of an alcohol beverage as a cause of or contributing to the achievement of sporting, sexual or other success [24], companies posted such content on their YouTube channels.

Furthermore, references to entrepreneurship and inspirational quotes on some of the Indian YouTube pages are likely to reflect the history of the caste system in India and a resulting focus on positioning alcohol products as livelihood opportunities for the disadvantaged strata of society. The equivalent Indian regulations does not include online advertisings so there is no mechanism to regulate the portrayal of such content and Indian brands can freely post videos based on these themes on their YouTube pages [23].

The above mentioned variations in marketing strategies illustrate the ability of brands to generate country-specific content for users to engage with and share. Internet advertising can also respond to users’ comments much more rapidly than traditional advertising, which is far less flexible [51]. In the Indian context, this flexibility allows content to be delivered in multiple languages to cater to different segments within the larger culture [52]. This approach was not evident on the Australian sites, despite high levels of multiculturalism within Australia. This is likely attributed to 77% of people in Australia who only speak English at home [53].

The study has several limitations that affect the interpretation of findings. First, the inclusion of only two countries in a cross-country comparison limits the generalizability of the study results to populations with different socio-cultural norms and characteristics. Also, the limited time period for data collection that could have different seasonal characteristics for each country means that other possible content categories could have emerged outside that period.

Second, there is likely to be some selection bias in the identified categories resulting from identifying the brands with the highest number of subscriptions rather than using a random selection from all eligible brands. Further, ‘astroturfing’, wherein alcohol marketers create multiple (fake) brand ambassadors on their social media websites makes their ‘real’ popularity uncertain [54]. This could hold true especially in the Indian context due to the potential socio-cultural inhibitions towards alcohol [20, 44], which could have inhibited ‘real’ users from engaging with brands. Also, we could not determine if users were unique subscribers to a single brand or to multiple brands, so the total number of subscribers is unknown.

Third, the data were collected during a discrete time period; however, the number of subscriptions accumulates over time. We were unable to track the number of new subscriptions in that period and also if they were related to the content posted during that time. Fourth, given the gender disparities in alcohol use in India [18], an analysis by gender would also be of interest; however users’ genders were not identifiable in the current dataset. Similarly, subscribers’ age-related information was not available and thus we were unable to identify the ages of those engaging with the content. However, the brands employed strategies such as competitions, prize draws, game-related apps, free tickets to music events, which together with existing evidence, links the popularity of these strategies to young people [8, 9, 32].

There is no effective age restriction when creating a YouTube account [https://support.google.com/youtube/answer/2802167?rd=1]. Although videos with content deemed inappropriate for minors are age-restricted [https://support.google.com/youtube/answer/2802167?rd=1], the content can still be viewed if users misrepresent their ages while creating their accounts. Barry et al. (2015, p.89) demonstrated that “every underage profile, regardless of age, was able to successfully subscribe to each of the 16 (100%) official YouTube channels”, in their study [33]. Building on the existing evidence [33], this study thus demonstrates the potential of brands to expose children and adolescents to alcohol marketing on YouTube. Due to deficiencies in the existing Australian alcohol advertising code (ABAC), the described marketing techniques breach this code. This warrants policymakers better regulating social media alcohol advertising, particularly in relation to younger social media users [5, 34, 55]. Further, as the Advertising Standard Council of India does not regulate online alcohol advertising [23], YouTube provides alcohol marketers with unfettered opportunities to promote their products. This calls for the formulation of policies regulating online alcohol content and their effective enforcement, in the Indian context.

Finally, this study undertook a largely exploratory approach to report the techniques utilized by alcohol brands to engage consumers on YouTube. As work on this topic is in its infancy, initial exploratory work was required to inform subsequent in-depth analysis. Additional cross-national comparisons of this kind and over different social media platforms are needed to understand how alcohol marketers adapt content in specific national contexts.

## Conclusions

This cross-national comparison demonstrates that YouTube provides alcohol marketers with an advertising platform where they utilize tailored marketing approaches to cater to specific national contexts and develop content on the cultural meanings users invoke in their interactions with these strategies. Those exposed to alcohol marketing on YouTube are likely to include those under the legal drinking age.

## Additional files

Additional file 1: User engagement with YouTube (February 2016 – March 2016). (DOC 53 kb)

Additional file 2: Types of strategies identified on the top 20 alcohol brands with the greatest YouTube presence, 10 each for India and Australia. (DOC 74 kb)

Additional file 3: References to the content posted on brands’ YouTube pages. (DOC 32 kb)

## Abbreviations

ABAC: Alcohol Beverages Advertising Code
ABS: Australian Bureau of Statistics
AIHW: Australian Institute of Health and Welfare
ASCI: Advertising Standard Council of India
IIPS: International Institute for Population Sciences
TESD: Time- and event-specific drinking
WHO: World Health Organization
