# Supplementary material for: Alcohol marketing on YouTube: exploratory analysis of content adaptation to enhance user engagement in different national contexts
Source: BMC Public Health. 2018 Jan 16;18:141. doi: 10.1186/s12889-018-5035-3 (PMC5771215; doi:10.1186/s12889-018-5035-3)
Supplement: Supplementary file 1 — User engagement with YouTube (February 2016 – March 2016). (DOC 53 kb) [file 12889_2018_5035_MOESM1_ESM.doc]

Additional file 1: User engagement with YouTube (February 2016 – March 2016)

| **Brand** | **Beverage Category** | **Year started on YouTube** | **Subscriptions** | **Views** | **Videos** |
| --- | --- | --- | --- | --- | --- |
| **India** | | | | | |
| McDowell’s No. 1 | Whisky | 2010 | 5,375 | 20,445,319 | 125 |
| Kingfisher | Beer | 2006 | 4,944 | 6,680,903 | 609 |
| Blenders Pride ^a^ | Whisky | 2011 | 1,359 | 3,967,600 | 128 |
| Bacardi ^b^ | Rum | 2010 | 1,001 | 1,834,536 | 51 |
| Foster's | Beer | 2013 | 475 | 1,011,470 | 5 |
| Haywards 5000 | Whisky | 2006 | 432 | 248,991 | 23 |
| White Mischief | Vodka | 2011 | 188 | 96,160 | 76 |
| Officer’s Choice | Whisky | 2013 | 60 | 27,270 | 4 |
| Breezer ^b^ | RTD | 2013 | 22 | 399,845 | 4 |
| Ricard ^a^ | Whisky | 2015 | 12 | 707 | 7 |
| **Subtotal** |  |  | **13,868** | **34,712,801** | **1,032** |
| **Australia** | | | | | |
| Jameson Irish Whiskey ^a^ | Whisky | 2007 | 40,724 | 31,927,010 | 207 |
| Absolut ^a^ | Vodka | 2006 | 16,562 | 17,504,284 | 230 |
| Bombay Sapphire ^b^ | Gin | 2006 | 8,198 | 3,048,482 | 120 |
| XXXX | Beer | 2005 | 6,881 | 3,242,233 | 271 |
| Corona Extra | Beer | 2010 | 6,476 | 4,935,963 | 68 |
| Coopers Ale | Beer | 2010 | 1,792 | 733,580 | 140 |
| Carlton Draught | Beer | 2006 | 1,417 | 12,268 | 3 |
| Bundaberg Rum | Rum | 2009 | 1,341 | 656,018 | 19 |
| Jacob's Creek | Wine | 2010 | 811 | 1,973,077 | 172 |
| Jack Daniel’s | Whisky | 2010 | 811 | 1,972,833 | 112 |
| **Subtotal** |  |  | **85,013** | **66,005,748** | **1,342** |
| **TOTAL** |  |  | **98,881** | **100,718,549** | **2,344** |

RTD = ready to drink (pre-mixed beverage)

^a^ Pernod Ricard; ^b^ Bacardi
