# Supplementary material for: Alcohol marketing on YouTube: exploratory analysis of content adaptation to enhance user engagement in different national contexts
Source: BMC Public Health. 2018 Jan 16;18:141. doi: 10.1186/s12889-018-5035-3 (PMC5771215; doi:10.1186/s12889-018-5035-3)
Supplement: Supplementary file 2 — Types of strategies identified on the top 20 alcohol brands with the greatest YouTube presence, 10 each for India and Australia. (DOC 74 kb) [file 12889_2018_5035_MOESM2_ESM.doc]

Additional file 2: Types of strategies identified on the top 20 alcohol brands with the greatest YouTube presence, 10 each for India and Australia

|  | **Indian Brands (listed in order of popularity by subscriptions)** | | | | | | | |
| --- | --- | --- | --- | --- | --- | --- | --- | --- |
|  | **McDowell’s No. 1** | **Kingfisher** | **Blenders Pride** | | **Bacardi** | | **Foster's** | |
| Type of content | - Music - Camaraderie - Competitions | - Music - TESD - Sexually suggestive content | - Fashion - Celebrity endorsements | | - Music - Sexually suggestive content - Memes - TESD - Cocktail recipes - Competitions | | - TESD - Music | |
| Link to official website | x | ✓ | ✓ | | x | | x | |
| Video advert | ✓ | ✓ | x | | x | | ✓ | |
| Link to Facebook | ✓ | ✓ | ✓ | | ✓ | | ✓ | |
| Link to Google+ | x | ✓ | ✓ | | ✓ | | ✓ | |
| Responsible drinking messages | x | x | x | | ✓ | | ✓ | |
| Legal drinking messages | ✓ | ✓ | x | | x | | x | |
|  | **Indian Brands (continued)** | | | | | | |  |
|  | **Haywards 5000** | **White Mischief** | **Officer’s Choice** | **Breezer** | | **Ricard** | |  |
| Type of content | - Competitions - Inspirational talks - Entrepreneurial programs | - TESD - Celebrity endorsements - Sexually suggestive content - Sports | - Fashion - Sexually suggestive content - Inspirational talks | - Cocktail recipes - Competitions - TESD | | - TESD | |  |
| Link to official website | ✓ | ✓ | x | ✓ | | x | |  |
| Video advert | ✓ | x | x | x | | x | |  |
| Link to Facebook | ✓ | ✓ | x | x | | x | |  |
| Link to Google+ | ✓ | ✓ | x | x | | x | |  |
| Responsible drinking messages | x | ✓ | x | x | | ✓ | |  |
| Legal drinking messages | x | ✓ | x | x | | x | |  |

|  | **Australian Brands (listed in order of popularity by subscriptions)** | | | | |
| --- | --- | --- | --- | --- | --- |
|  | **Jameson Irish Whiskey** | **Absolut** | **Bombay Sapphire** | **XXXX** | **Corona Extra** |
| Type of content | - Brand heritage - TESD - Togetherness/ Camaraderie - Food/ Cocktail recipes | - TESD - Competitions - Cocktail recipes | - TESD - Cocktail recipes | - Brand heritage - TESD - Food recipes | - Food recipes - TESD - Music |
| Link to official website | ✓ | ✓ | x | ✓ | ✓ |
| Video advert | ✓ | ✓ | x | x | ✓ |
| Link to Facebook | ✓ | ✓ | ✓ | x | x |
| Link to Google+ | ✓ | ✓ | ✓ | x | ✓ |
| Responsible drinking messages | ✓ | x | ✓ | x | x |
| Legal drinking messages | x | x | x | x | x |

|  | **Australian Brands (continued)** | | | | |
| --- | --- | --- | --- | --- | --- |
|  | **Coopers Ale** | **Carlton Draught** | **Bundaberg Rum** | **Jacob's Creek** | **Jack Daniel’s** |
| Type of content | - Memes - Food/ Cocktail recipes | - TESD - Sports - Fashion - Memes | - TESD - Food/ Cocktail recipes - Apps - Gender- specific posts | - Camaraderie - TESD - Brand heritage - Inspirational talks | - Competitions - Cocktail recipes - Memes - Music - Brand heritage |
| Link to official website | ✓ | x | x | ✓ | ✓ |
| Video advert | ✓ | x | ✓ | ✓ | ✓ |
| Link to Facebook | ✓ | ✓ | ✓ | ✓ | ✓ |
| Link to Google+ | ✓ | ✓ | x | x | x |
| Responsible drinking messages | x | x | ✓ | ✓ | ✓ |
| Legal drinking messages | x | x | x | ✓ | ✓ |

TESD = Time- and Event-Specific Drinking

✓=content present; x=content absent
