# Supplementary material for: Alcohol marketing on YouTube: exploratory analysis of content adaptation to enhance user engagement in different national contexts
Source: BMC Public Health. 2018 Jan 16;18:141. doi: 10.1186/s12889-018-5035-3 (PMC5771215; doi:10.1186/s12889-018-5035-3)
Supplement: Supplementary file 3 — References to the content posted on brands’ YouTube pages. (DOC 32 kb) [file 12889_2018_5035_MOESM3_ESM.doc]

Additional file 3: References to the content posted on brands’ YouTube pages

| **Content** | **Number of references (%)** | |
| --- | --- | --- |
|  | **India** | **Australia** |
| TESD | 64 (33) | 89 (46) |
| Memes | 2 (1) | 5 (3) |
| Music | 32 (17) | 15 (7) |
| Sports | 10 (5) | 11 (6) |
| Fashion | 6 (3) | 10 (6) |
| Camaraderie | 17 (8) | 12 (6) |
| Competitions | 20 (10) | 14 (7) |
| Sexually suggestive content | 19 (10) | 0 (0) |
| Consumption suggestions (food/cocktail recipes) | 14 (7) | 21 (11) |
| Inspirational talks | 8 (4) | 5 (3) |
| Brand heritage  **Total** | 0 (0)  **192 (100)** | 12 (6)  **194 (100)** |

TESD = Time- and Event-Specific Drinking
